# Diabetes mellitus in patients with heart failure and effect modification of risk factors for short-term mortality: An observational study from the *Registro Colombiano de Falla Cardíaca* (RECOLFACA)

**DOI:** 10.7705/biomedica.6951

**Published:** 2024-05-31

**Authors:** Luis Eduardo Echeverría, Clara Saldarriaga, Sebastián Campbell-Quintero, Lisbeth Natalia Morales-Rodríguez, Juan David López-Ponce de León, Andrés Felipe Buitrago, Erika Martínez-Carreño, Jorge Alberto Sandoval-Luna, Alexis Llamas, Gustavo Adolfo Moreno-Silgado, Julián Vanegas-Eljach, Nelson Eduardo Murillo-Benítez, Ricardo Gómez-Paláu, Alex Arnulfo Rivera-Toquica, Juan Esteban Gómez-Mesa

**Affiliations:** 1 Departamento de Cardiología, Fundación Cardiovascular de Colombia, Bucaramanga, Colombia Fundación Cardiovascular de Colombia Departamento de Cardiología Fundación Cardiovascular de Colombia Bucaramanga Colombia; 2 Departamento de Cardiología, Clínica Cardio VID, Medellín, Colombia Clínica Cardio VID Departamento de Cardiología Clínica Cardio VID Medellín Colombia; 3 Departamento de Cardiología, Clínica Medilaser, Florencia, Colombia Clínica Medilaser Departamento de Cardiología Clínica Medilaser Florencia Colombia; 4 Departamento de Cardiología, Clínica Medilaser, Neiva, Colombia Clínica Medilaser Departamento de Cardiología Clínica Medilaser Neiva Colombia; 5 Departamento de Cardiología, Fundación Valle de Lili, Cali, Colombia Fundación Valle de Lili Departamento de Cardiología Fundación Valle de Lili Cali Colombia; 6 Departamento de Cardiología, Fundación Santa Fe de Bogotá, Bogotá, D.C., Colombia Fundación Santa Fe de Bogotá Departamento de Cardiología Fundación Santa Fe de Bogotá Bogotá, D.C. Colombia; 7 Departamento de Cardiología, Institución Clínica Iberoamérica Sanitas, Barranquilla, Colombia Institución Clínica Iberoamérica Sanitas Departamento de Cardiología Institución Clínica Iberoamérica Sanitas Barranquilla Colombia; 8 Departamento de Cardiología, Cardiología Siglo XXI, Ibagué, Colombia Cardiología Siglo XXI Departamento de Cardiología Cardiología Siglo XXI Ibagué Colombia; 9 Departamento de Cardiología, Clínica Las Américas, Medellín, Colombia Clínica Las Américas Departamento de Cardiología Clínica Las Américas Medellín Colombia; 10 Departamento de Cardiología, Hospicardio, Montería, Colombia Hospicardio Departamento de Cardiología Hospicardio Montería Colombia; 11 Departamento de Cardiología, Hospital Alma Máter, Antioquia, Colombia Hospital Alma Máter Departamento de Cardiología Hospital Alma Máter Antioquia Colombia; 12 Departamento de Cardiología, Angiografía de Occidente, Cali, Colombia Angiografía de Occidente Departamento de Cardiología Angiografía de Occidente Cali Colombia; 13 Departamento de Cardiología, Clínica Imbanaco-Grupo Quirón Salud, Cali, Colombia Clínica Imbanaco-Grupo Quirón Salud Departamento de Cardiología Clínica Imbanaco-Grupo Quirón Salud Cali Colombia; 14 Departamento de Cardiología, Centro Médico para el Corazón, Pereira, Colombia Centro Médico para el Corazón Departamento de Cardiología Centro Médico para el Corazón Pereira Colombia; 15 Departamento de Ciencias de la Salud, Universidad Icesi, Cali, Colombia Universidad Icesi Departamento de Ciencias de la Salud Universidad Icesi Cali Colombia

**Keywords:** Diabetes mellitus, diabetes mellitus, type 2, heart failure, risk factors, mortality, Latin America, Colombia, diabetes mellitus, diabetes mellitus de tipo 2, insuficiencia cardiaca, factores de riesgo, mortalidad, América Latina, Colombia

## Abstract

**Introduction.:**

Heart failure and type 2 diabetes mellitus are critical public health issues.

**Objective.:**

To characterize the risk factors for mortality in patients with heart failure and type 2 diabetes mellitus from a large registry in Colombia and to evaluate the potential effect modifications by type 2 diabetes mellitus over other risk factors.

**Materials and methods.:**

Heart failure patients with and without type 2 diabetes mellitus enrolled in the *Registro Colombiano de Falla Cardíaca* (RECOLFACA) were included.

RECOLFACA enrolled adult patients with heart failure diagnosis from 60 medical centers in Colombia during 2017-2019. The primary outcome was all-cause mortality. Survival analysis was performed using adjusted Cox proportional hazard models.

**Results.:**

A total of 2514 patients were included, and the prevalence of type 2 diabetes mellitus was 24.7% (n = 620). We found seven independent predictors of short-term mortality for the general cohort, chronic obstructive pulmonary disease, sinus rhythm, triple therapy, nitrates use, statins use, anemia, and hyperkalemia. In the type 2 diabetes mellitus group, only the left ventricle diastolic diameter was an independent mortality predictor (HR = 0.96; 95% CI: 0.93-0.98). There was no evidence of effect modification by type 2 diabetes mellitus on the relationship between any independent predictors and all-cause mortality.

However, a significant effect modification by type 2 diabetes mellitus between smoking and mortality was observed.

**Conclusions.:**

Patients with type 2 diabetes mellitus had higher mortality risk. Our results also suggest that type 2 diabetes mellitus diagnosis does not modify the effect of the independent risk factors for mortality in heart failure evaluated. However, type 2 diabetes mellitus significantly modify the risk relation between mortality and smoking in patients with heart failure.

## Resumen

**Introducción.:**

La insuficiencia cardíaca y la diabetes mellitus de tipo 2 son problemas críticos de salud pública.

**Objetivo.:**

Caracterizar los factores de riesgo de mortalidad en pacientes con insuficiencia cardíaca y la diabetes mellitus de tipo 2 de un registro grande en Colombia y evaluar las posibles modificaciones del efecto de la diabetes mellitus de tipo 2 sobre otros factores de riesgo.

**Materiales y métodos.:**

Se incluyeron pacientes con insuficiencia cardíaca con y sin diabetes mellitus de tipo 2, inscritos en el Registro Colombiano de Insuficiencia Cardíaca (RECOLFACA). RECOLFACA incorporó pacientes adultos con diagnóstico de insuficiencia cardíaca de 60 centros médicos de Colombia durante 2017-2019. El resultado primario fue la mortalidad por todas las causas. El análisis de supervivencia se realizó utilizando modelos ajustados de riesgos proporcionales de Cox.

**Resultados.:**

Se incluyeron 2.514 pacientes, la prevalencia de diabetes mellitus de tipo 2 fue del 24,7 % (n = 620). Encontramos siete predictores independientes de mortalidad a corto plazo para la enfermedad pulmonar obstructiva crónica del grupo sin diabetes mellitus de tipo 2, el ritmo sinusal, la terapia triple, el uso de nitratos, el uso de estatinas, la anemia y la hiperpotasemia. En el grupo de diabetes mellitus de tipo 2, solo el diámetro diastólico del ventrículo izquierdo fue un predictor de mortalidad independiente (HR = 0,96; IC_95 %_: 0,93 - 0,98). No hubo evidencia de modificación del efecto de la diabetes mellitus de tipo 2 sobre la relación entre ningún predictor independiente y la mortalidad por todas las causas. Sin embargo, se observó una modificación significativa del efecto de la diabetes mellitus de tipo 2 entre el tabaquismo y la mortalidad.

**Conclusiones.:**

Los pacientes con diabetes mellitus de tipo 2 tuvieron mayor riesgo de mortalidad. Los resultados también sugieren que el diagnóstico de diabetes mellitus de tipo 2 no modifica el efecto de los factores de riesgo independientes de mortalidad en IC evaluados. Sin embargo, la diabetes mellitus de tipo 2 modifica significativamente la relación de riesgo entre mortalidad y tabaquismo en pacientes con insuficiencia cardíaca, posiblemente debido a un efecto sinérgico negativo que resulta en lesión vascular.

Heart failure and type 2 diabetes mellitus represent two of the most relevant non-transmissible chronic diseases worldwide, representing a critical public health issue nowadays ^1^. An estimated 6.2 million individuals were living with heart failure in the United States between 2013 and 2016, while its prevalence in Latin American countries was 1% [95% confidence interval (95%CI): 0.1%- 2.7%) between 1994 and 2014 ^2^^,^^3^. Moreover, approximately 26 million people are affected by heart failure worldwide, representing an estimated health expenditure of around USD $31 billion by 2012, which is expected to increase by 127% by 2030 ^3^. On the other hand, an estimated 462 million individuals have been diagnosed with type 2 diabetes mellitus by 2017, reflecting a prevalence rate of 6059 cases per 100,000 population. Moreover, around one million deaths per year can be attributed to diabetes alone, positioning this disease as the ninth leading cause of mortality worldwide ^4^.

Heart failure and type 2 diabetes mellitus commonly coexist, exponentially increasing the risk of complications in the patients affected by both conditions ^5^. This coexistence is derived from the common pathophysiological pathways both heart failure and type 2 diabetes mellitus share ^1^^,^^6^. This association was first observed in the Framingham study after more than 40 years of follow-up. In this cohort, men and women with diabetes mellitus had a two- and five-times higher risk of developing heart failure compared to the general population, respectively ^7^. These findings could partially explain why the prevalence of type 2 diabetes mellitus in heart failure patients is exceptionally high (estimated to be around 30% to 50%) ^5^. However, a new hypothesis suggesting a bidirectional relation between heart failure and type 2 diabetes mellitus has also been gaining relevance in recent years, giving further explanation to this observed prevalence, and highlighting the importance of type 2 diabetes mellitus and heart failure coexistence ^1^.

In this context, type 2 diabetes mellitus can modify heart failure’s disease course, increasing the risk of adverse outcomes, including rehospitalizations, prolonged hospital stays, and even mortality, mainly in the context of heart failure with reduced ejection fraction ^8^^,^^9^. Nonetheless, the knowledge of the potential interactions and effect modifications that type 2 diabetes mellitus can elicit on mortality-associated risk factors is still poorly understood ^10^. Understanding these is crucial for an optimal approach and adequate guiding of the patient’s management to achieve the expected goals ^11^. The present study aimed to characterize the risk factors for mortality in patients with heart failure and type 2 diabetes mellitus from RECOLFACA and evaluate the potential effect modifications by type 2 diabetes mellitus over other risk factors.

## Materials and methods

### Study design and population

The RECOLFACA is a prospective cohort study conducted at 60 medical centers, heart failure clinics, and cardiology outpatient centers in Colombia. Patient enrollment started in February 2017 and ended in October 2019. It included all adult patients (older than 18 years) with a clinical diagnosis of heart failure of any etiology based on the guideline recommendations at the time of inclusion, with at least one hospitalization due to heart failure in the 12 months before enrollment. Specific inclusion and exclusion criteria, along with additional methodologic characteristics of the registry, are described elsewhere ^12^^,^^13^.

### Data collection

Information regarding sociodemographic, clinical, and laboratory variables was registered at baseline. Type 2 diabetes mellitus diagnosis was based on self-report, laboratory test results (fasting glucose level > 125 mg/dl or glycated hemoglobin [HbA1c] of more than 7%), or glucose-lowering therapy use. Heart failure severity was assessed using the New York Heart Association (NYHA) classification.

An ischemic disease diagnosis was registered if the patient underwent a coronary revascularization procedure or if a previous myocardial infarction history was present. The following comorbidities were assessed: chronic kidney disease, arterial hypertension, atrial fibrillation, chronic obstructive pulmonary disease (COPD), thyroid disease, and dyslipidemia. The estimated glomerular filtration rate (eGFR) was calculated with the MDRD formula, and an eGFR < 60 ml/min/1.73 m^2^ was considered the cut-off for chronic kidney disease. Available data on additional echocardiographic variables was registered. The left ventricle ejection fraction variable was available from 2041 patients (81.2%), 502 from the type 2 diabetes mellitus group (80.9%) and 1539 from the non-type 2 diabetes mellitus group (81.3%), the systolic diameter of the left ventricle variable was available from 1305 patients (51.9%), 299 from the type 2 diabetes mellitus group (48.2%) and 1006 from the non-type 2 diabetes mellitus group (53.1%), and the valvular pathology variable was available from all patients. We considered triple therapy as the presence of the prescription of an angiotensin-converting enzyme inhibitor (ACEI) or angiotensin receptor blocker (ARB), plus a beta-blocker and a mineralocorticoid receptor antagonist.

### Outcomes

The primary outcome of the study was all-cause mortality. Data on this outcome was collected using a questionnaire applied by each heart failure clinic and center two times per year. The current results represent the data from the follow-up performed after six months of enrollment into the registry. Each center also reviewed each patient’s clinical records to assess specific data about the outcomes.

### Statistical analysis

At first, the total sample was divided into two groups (diabetics vs. nondiabetics). Baseline characteristics were described as medians and quartiles for quantitative variables, or absolute counts, proportions and percentages for categorical variables. Differences between groups were assessed using Pearson’s chi square and Fisher’s exact tests for categorical variables and Mann-Whitney U tests for quantitative variables. The cumulative incidence of the mortality events was calculated with their respective 95% confidence intervals. Survival analysis was performed using the Kaplan-Meier method and adjusted Cox proportional hazard models.

A multivariable Cox regression model including all the variables significantly associated with mortality was generated; after this, variables independently associated with this outcome were selected. The final model included all the independent predictors of mortality and was also adjusted by age, sex, chronic kidney disease diagnosis, NYHA classification, and left ventricle ejection fraction. Effect modification was assessed using multiple Cox regression and the Mantel-Haenszel method.

In summary, an effect modifier corresponds to a variable that differentially modifies a risk factor’s observed effect regarding a determined outcome. This results in different risk estimates between the evaluated groups when the effect modifier is present. A p value less than 0.05 (two-tailed test) was considered as statistically significant. All analyses were performed using statistical package Stata^™^, version 15 (Station College, Texas USA).

## Results

From the total 2528 patients included in RECOLFACA between February 2017 and October 2019, 2514 patients had information regarding type 2 diabetes mellitus diagnosis. The prevalence of type 2 diabetes mellitus among these patients was 24.6% (n = 620).

### Baseline characteristics

No significant demographic differences were observed between the groups regarding sex, age, and ethnicity (table 1). However, patients with type 2 diabetes mellitus diagnosis had a significantly higher prevalence of hypertension, coronary disease, valvular disease, chronic kidney disease, and dyslipidemia. On the other hand, non-diabetic individuals had more frequently a diagnosis of atrial fibrillation, valvular disease, and Chagas’ disease (table 1). Regarding the pharmacological therapy, patients with type 2 diabetes mellitus diagnosis were more frequently prescribed angiotensin receptor blockers, antiplatelets, and statins, while non-diabetics reported higher use of MRA, ACEI, and digoxin. Finally, diabetic patients showed a lower value of hemoglobin,and glomerular filtration rate while having a higher prevalence of electrolytic disorders.

**Table 1:** Sociodemographic and clinical characteristics of patients with heart failure diagnosis enrolled in the *Registro Colombiano de Insuficiencia Cardíaca* (RECOLFACA) by type 2 diabetes mellitus diagnosis

		Type 2 diabetes mellitus (N = 2514)			
		No (n = 1894) n (%)	Yes (n = 620) n (%)	Total n (%)	p value
Sex					0.195
	Female	790 (41.7)	277 (44.7)	1067 (42.4)	
	Male	1104 (58.3)	343 (55.3)	1447 (57.6)	
Age (years)		69 (59.78)	69 (62.77)	69 (59.78)	0.443
Race					0.400
	Asian	0 (0.0)	1 (0.2)	1 (0.0)	
	White	85 (4.5)	31 (5.0)	116 (4.6)	
	Indigenous	9 (0.5)	1 (0.2)	10 (0.4)	
	Hispanic	1398 (73.8)	455 (73.4)	1853 (73.7)	
	Mestizo	342 (18.1)	116 (18.7)	458 (18.2)	
	African-American	60 (3.2)	16 (2.6)	76 (3.0)	
Hypertension		1283 (67.7)	528 (85.2)	1811 (72.0)	< 0.001
Alcoholism		69 (3.6)	17 (2.7)	86 (3.4)	0.284
Cancer		75 (4.0)	26 (4.2)	101 (4.0)	0.797
Depression		34 (1.8)	13 (2.1)	47 (1.9)	0.630
Dementia		17 (0.9)	5 (0.8)	22 (0.9)	0.833
Coronary disease		482 (25.4)	224 (36.1)	706 (28.1)	< 0.001
COPD		328 (17.3)	113 (18.2)	441 (17.5)	0.606
Atrial fibrillation		456 (24.1)	104 (16.8)	560 (22.3)	< 0.001
Thyroid disease		279 (14.7)	109 (17.6)	388 (15.4)	0.088
Chronic kidney disease		257 (13.6)	177 (28.5)	434 (17.3)	< 0.001
Valvular disease		342 (18.1)	87 (14.0)	429 (17.1)	0.021
Smoking habits (former or current)		341 (18)	112 (18.1)	453 (18)	0.836
CABG		109 (5.8)	61 (9.8)	170 (6.8)	< 0.001
Dyslipidemia		427 (22.5)	220 (35.5)	647 (25.7)	< 0.001
Ischaemic heart disease		475 (25.1)	218 (35.2)	693 (27.6)	< 0.001
Chagas’ disease		76 (4.0)	12 (1.9)	88 (3.5)	0.015
NYHA					0.094
	I	241 (12.7)	57 (9.2)	298 (11.9)	
	II	998 (52.7)	352 (56.8)	1350 (53.7)	
	III	565 (29.8)	182 (29.4)	747 (29.7)	
	IV	90 (4.8)	29 (4.7)	119 (4.7)	
ACC/AHA classification					
	C	1792 (94.6)	585 (94.4)	2377 (94.6)	0.805
	D	102 (5.4)	35 (5.7)	137 (5.5)	
ACEI		674 (35.6)	172 (27.7)	846 33.7)	< 0.001
ARB		766 (40.4)	303 (48.9)	1069 (42.5)	< 0.001
Diuretics		1257 (66.4)	436 (70.3)	1693 (67.3)	0.068
Beta-blockers		1639 (86.5)	550 (88.7)	2189 (87.1)	0.162
ARNI		176 (9.3)	69 (11.1)	245 (9.7)	0.181
MRA		1082 (57.1)	317 (51.1)	1399 (55.6)	0.009
Ivabradine		111 (5.9)	39 (6.3)	150 (5.9)	0.790
Digoxin		206 (10.9)	45 (7.3)	251 (10.0)	0.009
Nitrates		62 (3.3)	29 (4.7)	91 (3.6)	0.104
Antiagregants		804 (42.4)	356 (57.4)	1160 (46.1)	< 0.001
Statins		984 (52.0)	407 (65.6)	1391 (55.3)	< 0.001
Anticoagulants		506 (26.7)	137 (22.1)	643 (25.6)	0.022
Pacemaker					0.975
Dual-chamber		73 (3.9)	25 (4.0)	98 (3.9)	
Single-chamber		37 (2.0)	12 (1.9)	49 (2.0)	
Other implantable devices					0.826
	ICD	286 (15.1)	84 (13.6)	370 (14.7)	
Resynchronization therapy		35 (1.8)	13 (2.1)	48 (1.9)	
ICD + resynchronization therapy		98 (5.2)	29 (4.7)	127 (5.1)	
Sinus rhythm		636 (33.6)	231 (37.3)	867 (34.5)	0.094
QRS complex					
	< 120 ms	633 (33.4)	200 (32.3)	833 (33.1)	0.785
	> 120 ms	42 (2.2)	17 (2.7)	59 (2.3)	
LV diastolic diameter		57 (48-65)	55 (48-63)	57 (48-65)	0.045
LVEF		32 (25-42)	33 (25-42)	33 (25-42)	0.645
Hemoglobin (mg/dl)		13 (12.14)	12 (11.14)	13 (12.14)	< 0,001
Anemia		494 (35.4)	232 (48.3)	726 (38.7)	< 0,001
Serum creatinine		1.1 (0.9-1.35)	1.2 (0.9-1.6)	1.1 (0.9-1.4)	< 0,001
GFR (ml/min/1,73 m^3^)		59 (44.78)	53 (36.74)	57 (43.77)	< 0,001
Hyponatremia		1079 (56.9)	390 (62.9)	240 (9.5)	< 0,001
Hyperkalemia		113 (8.3)	63 (12.6)	176 (9.4)	0,005
NT-proBNP		2151 (855-5089)	2795 (1002-6857)	2255 (950-5594)	0,101

COPD: Chronic obstructive pulmonary disease; CABG: Coronary artery bypass grafting; NYHA: New York Heart Association; ACC/AHA: American College of Cardiology/American Heart Association; ACEI: Angiotensin-converting enzyme inhibitor; ARB: Angiotensin receptor blockers; ARNI: Angiotensin receptor-neprilysin inhibitor; MRA: Mineralocorticoid receptor antagonist; ICD: Implantable cardioverter defibrillator; LV: Left ventricle; LVEF: Left ventricular ejection fraction; GFR: Glomerular filtration rate; NT-proBNP: N-terminal pro b-type natriuretic peptide

### Mortality and associated factors

The median follow-up time was 215 days (Q_1_: 188; Q_3_: 254). A total of 170 patients (6.76%) died during the follow-up, for a mortality rate of 0.29 per 1,000 person-years (95% CI: 25.4-34.5). Significantly higher mortality was observed in the type 2 diabetes mellitus group than in the non-diabetic group (8.9% vs. 6.1%, respectively; p = 0.016). Supplementary table 1 summarizes the association between the evaluated variables and mortality use a bivariate analysis. From these variables, we included those that were significantly associated with mortality in the multivariable Cox regression model. Finally, after adjusting by age, sex, type 2 diabetes mellitus, bchronic kidney disease, NYHA classification, and left ventricle ejection fraction, seven independent predictors of short-term mortality were identified (table 2).

**Table 2:** Factors independently associated with short-term mortality in the current cohort of heart failure patients. The logistic regression model was also adjusted by age, sex, chronic kidney disease, New York Heart Association classification, and left ventricular ejection fraction.

Factor	Hazard ratio (95% CI)	P value
COPD	1.76 (1.03-3.02)	0.039
Sinus rhythm	0.56 (0.34-0.92)	0.022
Triple therapy	0.41 (0.23-0.73)	0.003
Nitrates use	3.01 (1.19-7.63)	0.020
Statins use	0.48 (0.28-0.80)	0.005
Anemia	1.85 (1.12-3.04)	0.016
Hyperkalemia	3.09 (1.64-5.83)	0.001

CI: Confidence interval; COPD: Chronic obstructive pulmonary disease

Furthermore, we also analyzed the variables associated with mortality in the type 2 diabetes mellitus subgroup (table 3). In this specific sub-group, only chronic kidney disease, smoking status, statins use, anticoagulants use, left ventricle diastolic diameter, and anemia were significantly associated with the mortality outcome, being some of these factors different from those observed in the general cohort. The multivariate analysis showed that only the left ventricle diastolic diameter was an independent mortality predictor in the type 2 diabetes mellitus group (HR = 0.96; 95% CI: 0.93-0.98).

**Table 3:** A bivariate analysis evaluating the association between the sociodemographic and clinical variables with short-term all-cause mortality in patients with type 2 diabetes mellitus diagnosis of the *Registro Colombiano de Insuficiencia Cardíaca* (RECOLFACA)

		Alive (n = 565) n (%)	Dead (n =55) n (%)	Total (N = 620) n (%)	p value
Sex					
	Female	250 (44.25)	27 (49.09)	277 (44.68)	0.490
	Male	315 (55.75)	28 (50.91)	343 (55.32)	
Age (years)		69 (62.77)	69 (63.79)	69 (62.77)	0.453
Race					
	Asian	1 (0.18)	0 (0)	1 (0.16)	
	White	29 (5.13)	2 (3.64)	31 (5)	
	Indigenous	1 (0.18)	0 (0)	1 (0.16)	0.167
	Hispanic	420 (74.34)	35 (63.64)	455 (73.39)	
	Mestiza	102 (18.05)	14 (25.45)	116 (18.71)	
	African-American	12 (2.12)	4 (7.27)	16 (2.58)	
Hypertension		478 (84.60)	50 (90.91)	528 (85.16)	0.209
Current alcoholism		14 (2.48)	3 (5.45)	17 (2.74)	0.197
Cancer		21 (3.72)	5 (9.09)	26 (4.19)	0.058
Coronary disease		200 (35.40)	24 (43.64)	224 (36.13)	0.225
COPD		99 (17.52)	14 (25.45)	113 (18.23)	0.146
Atrial fibrillation		92 (16.28)	12 (21.82)	104 (16.77)	0.294
Thyroid disease		96 (16.99)	13 (23.64)	109 (17.58)	0.216
Chronic kidney disease		153 (27.08)	24 (43.64)	177 (28.55)	0.009
Valvular disease		80 (14.16)	7 (12.73)	87 (14.03)	0.770
Smoking habits (former or current)		96 (16.99)	16 (29.09)	112 (18.06)	0.026
CABG		57 (10.09)	4 (7.27)	61 (9.84)	0.503
Dyslipidemia		206 (36.46)	14 (25.45)	220 (35.48)	0.103
Chagas disease		11 (1.95)	1 (1.82)	12 (1.94)	0.947
NYHA					
	I	54 (9.56)	3 (5.45)	57 (9.19)	
	II	325 (57.52)	27 (49.09)	352 (56.77)	0.167
	III	162 (28.67)	20 (36.36)	182 (29.35)	
	IV	24 (4.25)	5 (9.09)	29 (4.68)	
ACC/AHA classification					
	C	533 (94.34)	52 (94.55)	585 (94.35)	0.949
	D	32 (5.66)	3 (5.45)	35 (5.65)	
Triple therapy		242 (42.83)	24 (43.64)	266 (42.90)	0.908
Diuretics		391 (69.20)	45 (81.82)	436 (70.32)	0.051
Digoxin		40 (7.08)	5 (9.09)	45 (7.26)	0.583
Nitrates		24 (4.25)	5 (9.09)	29 (4.68)	0.104
Antiplatelet		328 (58.05)	28 (50.91)	356 (57.42)	0.306
Statins		380 (67.26)	27 (49.09)	407 (65.65)	0.007
Anticoagulants		117 (20.71)	20 (36.36)	137 (22.10)	0.008
Pacemaker					0.054
	Dual-chamber	24 (4.25)	1 (1.81)	25 (4.42)	
	Single-chamber	9 (1.59)	3 (5.45)	12 (2.12)	
Other implantable devices					0.181
	ICD	94 (16.64)	12 (21.82)	106 (17.10)	
Resynchronization therapy		10 (1.77)	3 (5.45)	13 (2.10)	
ICD + resynchronization therapy		27 (4.78)	2 (3.62)	29 (4.68)	
Sinusal rhythm		213 (68.93)	18 (72)	231 (69.16)	0.749
Prolonged QRS complex		126 (40.78)	8 (32)	134 (40.12)	0.389
LV diastolic diameter		55 (48.63)	50.5 (3958)	55 (4863)	0.015
LVEF		34 (25.43)	28 (2040)	33 (2542)	0.101
Anemia		212 (48.51)	20 (46.51)	232 (48.33)	0.007
Serum creatinine		1.2 (0.93-1.60)	1.29 (1.1.8)	1.2 (0.95-1.61)	0.409
GFR (ml/min/1,73 m^3^)		53.35 (36.05-75.24)	52.37 (33.05-66.58)	53.3 (35.71-74.21)	0.521
Hyponatremia		61 (14.59)	9 (21.43)	70 (15.22)	0.240
Hyperkalemia		54 (11.82)	9 (20.45)	63 (12.57)	0.099
NT-proBNP		2790 (1099-5896)	9496 (996- 1671)	2795 (1002-6857)	0.254

COPD: Chronic obstructive pulmonary disease; CABG: Coronary artery bypass grafting; NYHA: New York Heart Association; ACC/AHA: American College of Cardiology/American Heart Association; ACEI: Angiotensin-converting enzyme inhibitor; ARB: Angiotensin receptor blockers; ARNI: Angiotensin receptor-neprilysin Inhibitor; MRA: Mineralocorticoid receptor antagonist; ICD: Implantable cardioverter defibrillator; LV: Left ventricle; LVEF: Left ventricular ejection fraction; GFR: Glomerular filtration rate; NT-proBNP: N-terminal pro b-type natriuretic peptide

### Interaction and effect modification by diabetes mellitus

There were relevant differences when assessing the independent factors by the type 2 diabetes mellitus group. At first, patients receiving triple medical therapy in the non-type 2 diabetes mellitus group had significantly lower mortality than those not receiving this therapeutic scheme.

**Figure 1:**
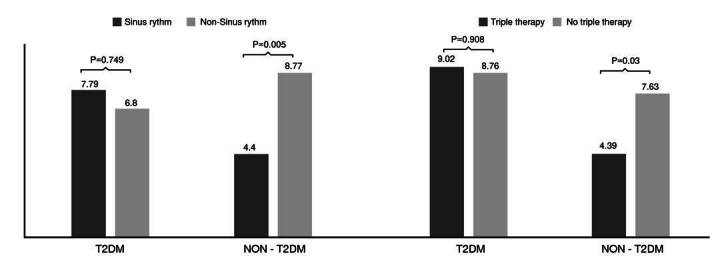
Mortality in patients according to sinus rhythm and triple therapy [angiotensin-converting enzyme inhibitor (ACEI)/angiotensin receptor blocker (ARB), beta-blocker and mineralocorticoid receptor antagonist (MRA)] by type 2 diabetes mellitus group.

On the other hand, the incidence of mortality was not statistically different in the group of patients with type 2 diabetes mellitus (figure 1). A similar result was observed regarding the report of sinus rhythm in the last electrocardiogram performed. No additional significant differences were observed for the other independent predictor variables.

**Figure 2:**
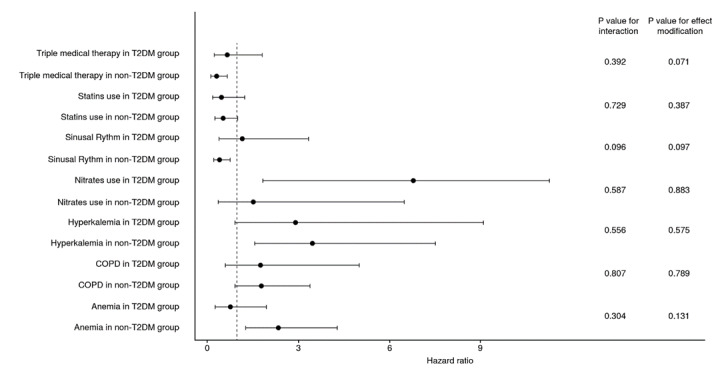
Adjusted association of each independent predictor by type 2 diabetes mellitus group and all-cause mortality. The p value for effect modification was calculated using the Mantel-Haenszel test of homogeneity.

Despite these findings, there was no evidence of effect modification by type 2 diabetes mellitus on the relationship between any of the independent predictors and all-cause mortality (figure 2). Furthermore, no interaction terms by type 2 diabetes mellitus were observed in the assessed sample (figure 2). Nevertheless, an interesting result was observed when assessing smoking history. Although it was not an independent predictor of short-term mortality in the general cohort, patients with type 2 diabetes mellitus and tobacco consumption (current or former) had a significantly higher risk of mortality (HR = 1.84; 95% CI: 1.01-3.35) while the difference was not statistically significant in non-diabetic individuals (HR = 0.61; 95% CI: 0.35-1.11). Moreover, a significant effect modification by type 2 diabetes mellitus on the association between tobacco consumption and mortality was observed (p value = 0.005), along with a significant interaction term between both variables (p value for interaction = 0.010).

## Discussion

In the present study, the differential characteristics of patients with heart failure with and without type 2 diabetes mellitus enrolled in the RECOLFACA were described. Almost one-fourth of the patients with heart failure from the registry had a concomitant diagnosis of type 2 diabetes mellitus, highlighting a lower use of MRA, ACEI, and digoxin in these patients, along with a higher prevalence of chronic kidney disease, anemia, and electrolytic disorders. Finally, type 2 diabetes mellitus had a higher risk of mortality in the univariate analysis. Only COPD, nitrate use, anemia, and hyperkalemia were independently associated with higher mortality risk in the general cohort. In contrast, sinus rhythm, triple therapy with ACEI/ARB, MRA and beta-blockers, and statin use were associated with a lower risk of this outcome. Regarding the type 2 diabetes mellitus group, only left ventricle diastolic diameter was independently associated with mortality.

Finally, in our registry, type 2 diabetes mellitus diagnosis did not modify the effect of independent predictors of all-cause mortality in patients with heart failure after adjusting by relevant sociodemographic and clinical variables. Likewise, several studies have evaluated the potential effect modification that type 2 diabetes mellitus could exert in different contexts, but only some of those performed in cardiovascular disease settings have reported that type 2 diabetes mellitus does not modify mortality risk when several risk factors are present ^10^^,^^14^^,^^15^. The study by Selvarajah *et al.*^10^, which evaluated patients with cardiovascular disease, observed that type 2 diabetes mellitus diagnosis did not modify the effect of renal impairment with all-cause mortality after a follow-up of 33.198 person-years. Similarly, Gerstein *et al.*^14^ observed that microalbuminuria was an independent risk factor for all-cause mortality both in patients with and without type 2 diabetes mellitus diagnosis. On the other hand, Ahmed *et al.*^15^ reported that type 2 diabetes mellitus modified the effect of eGFR regarding all-cause mortality risk; however, their analysis was not adjusted by albuminuria, which precluded a more precise assessment due to the impact of albuminuria in the risk of mortality.

In regard to the higher mortality found in the type 2 diabetes mellitus group when compared to non-diabetics, our results coincide with those reported in the meta-analysis performed by Dauriz *et al.*^16^, where they found approximately 30% increased risk of all-cause death. Moreover, they reported that nearly one-quarter of the patients with heart failure had diabetes, similar to what we reported in the RECOLFACA. The AMERICCAASS is a recent registry that also included heart failure patients older than 18 years, that reported a similar finding of 28% patients with heart failure and diabetes in their results from the first 1,000 patients enrolled from different Latin American countries, including Colombia ^17^. Another large registry that analyzed data from more than 45,000 patients from different continents, including Latin America, was the Reduction of Atherothrombosis for Continued Health (REACH) registry, where approximately 30% of increased mortality in diabetic group vs. non-diabetics was also found. However, the inclusion criteria of the REACH registry are different from ours as they not only included patients older than 18 years with heart failure, but all patients > 45 years old that had established atherosclerosis or ≥ 3 risk factors for atherosclerosis, hence, the results are not comparable to ours.

In our study patients with type 2 diabetes mellitus and tobacco consumption had a significantly higher risk of mortality. A related finding was reported by the study of Jeong *et al.*^18^ where they analyzed data from 349,137 type 2 diabetes mellitus Korean patients with current or history of smoking. In their study they reported that smoking cessation was associated with a 10% lower risk of all-cause mortality. However, contrary to our study, the population they studied did not have heart failure as a baseline condition, hence, our results are not completely comparable. This could suggest that the higher risk of mortality seen between patients with type 2 diabetes mellitus and tobacco consumption (current or former), has no direct relation to the heart failure diagnosis they had.

Our results regarding the variables that are independently associated with higher mortality risk in the general cohort coincide with previous reports. Although few studies have analyzed the prognosis of patients with heart failure and COPD, it has been reported as an independent predictor of death and heart failure hospitalization when reported in multivariable models ^19^. Only one study has explored the causes of increased mortality ^20^.

According to a study with 4606 acute care patients with congestive heart failure, the use nitrates is associated with increased relative risk of in-hospital mortality ^21^. Anemia has been found as an independent predictor by multiple studies, including the study by Gupta *et al.*^22^ where they found that anemia emerged as an independent predictor of all-cause mortality and heart failure hospitalizations at the end of the follow-up, and the recent study by Köseoğlu *et al.*^23^ who found similar results. Lastly, hyperkalemia is a known risk factor for mortality among critically ill patients and cardiac patients ^24^.

The systematic review of Girerd *et al.*^25^ showed that the benefits of heart failure treatment appear to be similar in patients with type 2 diabetes mellitus as in non-diabetic patients, suggesting a lack of effect modification by this condition.

However, Kroon *et al.*^26^ assessed the potential effect modification by type 2 diabetes mellitus in the association between B-type natriuretic peptide (BNP) and changes in left ventricular function markers in patients with incipient heart failure. In this study, type 2 diabetes mellitus modified BNP’s effect over left ventricular mass index, left atrial volume * left ventricular mass index, and E/e’ ratio, even after adjustment by sex, age, baseline left ventricular mass index, body mass index, and use of antihypertensives.

Finally, Ebong *et al.*^27^ reported that type 2 diabetes mellitus, independently of its treatment and severity, modified the effect of the association between lipid fractions and incident heart failure, potentially due to the pathophysiological process of glucolipotoxicity. Although in our study type 2 diabetes mellitus was identified as a significant effect modifier of the impact of smoking habits and mortality, there are no previous reports on this specific topic. However, it is worth mentioning that cigarette smoking has been identified as a contributor to all-cause mortality the general population, which is expected to be similar in type 2 diabetes mellitus patients ^28^. Moreover, among type 2 diabetes mellitus patients, cigarette smoking may accelerate cardiovascular disease mortality ^29^^,^^30^.

Regarding treatment, for several years, there were concerns about the use of beta-blockers in patients with type 2 diabetes mellitus due to the perceived risk of hypoglycemia, limiting their use in patients with heart failure and type 2 diabetes mellitus despite the benefit observed in heart failure trials ^25^^,^^31^. Furthermore, several clinical trials have reported that the impact of heart failure medical therapy on prospective outcomes in patients with type 2 diabetes mellitus could be significantly different from the one observed in non-diabetic patients ^32^. Unfortunately, some of these trials were performed in the 80s and 90s; thus, these studies did not assess interactions and effect modifications.

A recent post-hoc analyses and meta-analyses have suggested that the efficacy of the therapy with ACEI/ARB, MRA, and beta-blockers is similar in heart failure patients with and without type 2 diabetes mellitus, observations that are consistent with the results of the present study ^33^^-^^36^. These findings’ relevance lies in the possibility of promoting an optimal medical treatment for patients with heart failure despite being diabetic or not ^37^. To achieve this goal, non-cardiologists who treat patients with heart failure and type 2 diabetes mellitus should be invited to actively participate in the therapeutic optimization process and patient referral to specialized centers for interventional strategies. The involvement of nurses, general practitioners, internal medicine specialists, endocrinologists, and diabetologists in the process of up-titration of heart failure medications has been shown to be safe and efficient in achieving target doses of ACEI/ARB, MRA, and beta-blockers ^36^^-^^38^.

Finally, our findings showed that several comorbidities and clinical conditions were independently associated with a higher risk of short-term mortality in heart failure patients, which is consistent with the literature ^39^^-^^42^. The effect of these conditions on short term mortality risk was not modified by type 2 diabetes mellitus; nonetheless, we observed a significant effect modification by type 2 diabetes mellitus in the association between smoking status and mortality. This finding may derive from the common pathophysiological processes that both type 2 diabetes mellitus and smoking promote, which results in a higher incidence of macrovascular and microvascular complications due to a synergistic negative effect of these two conditions combined ^43^^,^^44^.

### Limitations of the study

The present study had from several limitations. The RECOLFACA does not collect information regarding HbA1C levels or antidiabetic treatment; therefore, adjustment by these important variables was not possible. Moreover, no information was available on the duration and severity of type 2 diabetes mellitus, including organ involvement. In addition, only a short follow-up was available for the analyses, therefore limiting the precision of the calculated estimates. Lack of power could have been an issue in the present study, potentially leading to false non-significance when the interaction terms and effect modifications were assessed. Moreover, several potential sources of variation were not accounted for in the present study such as body mass index or other relevant diagnoses like asthma. Finally, it was not possible to have data from all patients regarding echocardiographic variables which could be a confounding factor. Therefore, our results should be interpreted with caution.

RECOLFACA is the largest multicentric registry from Colombian patients with heart failure. In this registry, patients with type 2 diabetes mellitus were less frequently treated with MRA, ACEI, and digoxin than non-diabetics while having a higher mortality rate. Moreover, several clinical conditions were independently associated with mortality in this registry. Our results also suggest that type 2 diabetes mellitus diagnosis does not modify the effect of the independent risk factors for mortality in heart failure evaluated. However, type 2 diabetes mellitus was observed to significantly modify the risk relation between mortality and smoking in patients with heart failure.

*Competency in patient care and procedural skills:* patients with heart failure and type 2 diabetes mellitus in Colombia are less frequently treated with important heart failure drugs compared to non-diabetics, highlighting the need of optimizing the pharmacological therapy in this population. Finally, although type 2 diabetes mellitus did not modify the effect of mortality risk factors in heart failure, it seems it can elicit a relevant effect modification in the relationship between smoking history and mortality.

*Translational outlook:* Further research is needed to assess the role of tipe 2 diabetes mellitus as an effect modifier for risk factors of all-cause mortality in heart failure patients.
